# Associations between Mediterranean Diet Adherence, Quality of Life, and Mental Health in Patients with Multiple Sclerosis: A Cross-Sectional Study

**DOI:** 10.3390/jpm14020199

**Published:** 2024-02-11

**Authors:** Antonios Dakanalis, Christina Tryfonos, Eleni Pavlidou, Konstantinos Vadikolias, Sousana K. Papadopoulou, Olga Alexatou, Theofanis Vorvolakos, Maria Chrysafi, Dimitrios Fotiou, Maria Mentzelou, Aspasia Serdari, Maria Chatzidimitriou, Sophia Dimoliani, Gerasimos Tsourouflis, Constantinos Giaginis

**Affiliations:** 1 Department of Mental Health, Fondazione IRCCS San Gerardo dei Tintori, Via G.B. Pergolesi 33, 20900 Monza, Italy; antonios.dakanalis@unimib.it; 2 Department of Medicine and Surgery, University of Milan Bicocca, Via Cadore 38, 20900 Monza, Italy; 3 Department of Food Science and Nutrition, School of Environment, University of the Aegean, 81400 Lemnos, Greece; ch.trifon@aegean.gr (C.T.); elenpav@aegean.gr (E.P.); fnsd23003@fns.aegean.gr (O.A.); fnsm22030@fns.aegean.gr (M.C.); fnsd22007@fns.aegean.gr (M.M.); sdem@aegean.gr (S.D.); 4 Department of Neurology, School of Medicine, Democritus University of Thrace, 68100 Alexandroupolis, Greece; kvadikol@med.duth.gr; 5 Department of Nutritional Sciences and Dietetics, School of Health Sciences, International Hellenic University, 57400 Thessaloniki, Greece; sousana@the.ihu.gr; 6 Department of Psychiatry, School of Medicine, Democritus University of Thrace, 68100 Alexandroupolis, Greece; tvorvola@med.duth.gr (T.V.); aserntar@med.duth.gr (A.S.); 7 Department of Neurology, School of Medicine, Aristoteleio University of Thessaloniki, 54124 Thessaloniki, Greece; dfotiou@med.auth.gr; 8 Department of Biomedical Sciences, School of Health Sciences, International Hellenic University, 57400 Thessaloniki, Greece; chdimitr@ihu.gr; 9 Second Department of Surgery, Propedeutic, National and Kapodistrian University of Athens, Laikon General Hospital, 11527 Athens, Greece; gtsourouflis@med.uoa.gr

**Keywords:** multiple sclerosis, disease disability, quality of life, physical activity, serum biomarkers, Mediterranean diet, malnutrition, obesity, neurodegenerative diseases, symptoms’ severity

## Abstract

Background: The Mediterranean diet (MD) is well-known as a diet which may exert a protective effect against neurodegenerative diseases, including multiple sclerosis (MS). To date, only a few clinical surveys have assessed the potential effects of the MD in patients with MS. The purpose of the present study is to evaluate the potential effects of MD compliance on disease disability, quality of life, physical activity, depressive symptomatology, and blood biochemical parameters related to nutritional status in MS patients, considering several socio-demographic, anthropometric, and lifestyle characteristics. Methods: This is a cross-sectional study conducted on 558 adults with MS aged 18–64 years. Relevant questionnaires were utilized to evaluate socio-demographic and anthropometric parameters, disease disability (Expanded Disability Status Scale, EDSS), multidimensional health-related quality (MS Quality of Life-54, MSQOL-54), physical activity levels (International Physical Activity Questionnaire, IPAQ), depression (Beck Depression Inventory II, BDI-II), and MD adherence (MedDietScore), while several blood biochemical parameters were retrieved from the patients’ medical records. Results: Enhanced MD compliance was independently associated with a decreased frequency of overweight/obesity, as well as abdominal obesity, in patients suffering from MS. Elevated MD compliance was also independently associated with a decreased incidence of advanced disease disability, a higher prevalence of elevated physical activity, an improved quality of life, and lower depressive symptoms, as well as higher levels of certain blood biochemical parameters, which are effective indicators of iron deficiency and malnutrition. Conclusions: The present study found that higher MD adherence may slow down disease disability, promoting a better quality of life and mental health in adults with MS. Future prospective surveys are required to obtain conclusive results.

## 1. Introduction

Multiple sclerosis (MS) is a chronic inflammatory and degenerative disease of the central nervous system (CNS). MS constitutes one of the most common causes of non-traumatic disability in early adulthood, which can result in a variety of sensorimotor, cognitive, visual, and autonomous function symptoms, worsening at a significant level the daily quality of life of MS patients [1,2]. MS disease has been associated with deleterious physical and cognitive disturbances and a substantial decline in the performance status and functionality of MS patients [3]. The potential underlying causes of the permanent neurological deficits in MS patients have mainly been ascribed to axonal loss [4]. More to the point, demyelinated axons are sensitive to impairment because of the absence of trophic care by myelin sheaths and oligodendrocytes and the elevated susceptibility to immunology-related attacks [3,4]. The most common MS symptoms include paresthesia, numbness or faintness of the limbs, diplopia, vision damage, ataxia, weakness, bowel or bladder dysfunction, spasticity, and mental impairments [5,6]. During the progression of the disorder, these symptoms become more prominent, impeding the activities of day-to-day living and deteriorating the quality of life [6,7].

Novel treatment approaches have been developed to promote longevity in MS patients. Notably, Bruton tyrosine kinase (BTK) suppressors, a modern kind of immunoregulatory therapeutic approach, have emerged as a promising approach to slow down adverse disorder complications [8,9]. Moreover, novel attention has been attracted by the Epstein–Barr virus (EBV) for triggering MS progression [8,9]. However, the overall mortality rates associated with MS remain unaltered over time [10,11]. Remarkably, MS prevalence is gradually increasing, especially in women [10,11]. Thus, MS is currently considered the most frequent demyelinating disorder of the CNS with about 2.5 million individuals suffering from this disease globally [6,12].

MS diagnosis is typically established using the McDonald criteria that assess the occurrence of demyelinating alterations. Moreover, the combination of clinical assessment, neurological examination, medicine imaging, and spinal fluid examination is frequently implemented [13,14]. Disease disability is mainly evaluated using the Kurtzke Expanded Disability Status Scale (EDSS), which concerns both the neurological and functional characteristics of the disorder [15,16]. EDSS has been identified as the most widely used tool to reliably measure disease outcomes in clinical studies involving MS patients [17]. Even though the basic reasons for MS are still unclear, it has recently been considered an autoimmune disease [18]. Slight familial predispositions, geographical vulnerability, and virus infections (e.g., Epstein–Barr) were identified as possible risk factors. MS is frequent in early adulthood, females, smokers, persons with Epstein–Barr virus, obese individuals, and people who live below the equator [19,20].

Psychiatric symptomatology is commonly observed in individuals suffering from MS. Depressive symptoms show an elevated incidence in individuals suffering from MS compared to the general population [21]. Identifying the pathological progression governing these settings may result in the enhancement of various psychological batteries and scales to effectively recognize and manage depression symptomatology [22]. Mental disturbance symptomatology is a frequent comorbidity in MS, contributing to the disorder’s progression and influencing patients’ adherence to standard therapeutic remedies [23,24]. Despite their significance, the impacts of psychiatric disorders still remain unknown in individuals with MS [25]. Treating mental disturbances such as depression is critical to promote mental health, which could additionally enhance the quality of life of individuals with MS [26].

To date, the role of diet in MS has not been comprehensively investigated. Nutrition could be considered as a potential and effective preventive co-factor, negatively affecting the inflammation cascade by implicating both its molecular mechanisms and the intestinal microbiome in MS patients [27,28,29]. There are also several pieces of evidence that nutritional status can exert a crucial impact against neurodegenerative diseases, including MS [27,28,29]. Notably, individuals with MS follow a more restricted diet compared with healthy individuals, while iron levels are significantly decreased in individuals with MS, with the most decreased levels noted in patients with advanced stage MS [30]. A careful nutritional assessment of individuals with neurodegenerative diseases, as well as appropriate nutritional intervention and support, are highly fundamental in monitoring their disease [27,28,29]. Notably, there are several studies which indicate favorable health impacts for energy-restricted/intermittent fasting diets, a ketogenic diet, and a modified paleolithic diet. However, the above nutritional interventions cannot be applied for a long duration as they could lead to diverse nutrient deficiencies [31,32]. Moreover, no clinical study thus far has reported any potential beneficial impact of low-fat diets, gluten-free diet, and the Mediterranean-DASH Intervention for Neurodegenerative Delay (MIND) diet in patients with MS [31,32]. A network meta-analysis showed that several nutritional interventions could decrease MS-associated fatigue and enhance several mental and physical aspects of quality of life [33]. Nevertheless, the network meta-analysis has certain limitations due to the decreased quality of the analyzed clinical studies, and therefore these results need to be further proven by well-designed, randomized, controlled trials (RCTs) [33].

There are only a few pieces of evidence which have shown that an increased consumption of saturated fat may increase MS risk, while epidemiological studies suggested that unsaturated fatty acids could have a favorable impact on disease progression [34,35]. Several dietary supplements may be considered to slow down inflammatory effects and fatigue, enhancing autoimmune resistance in individuals with MS, and therefore enhancing quality of life and life expectancy [36,37]. However, no clinical recommendations for utilizing nutritional supplementations as co-therapeutic approaches against MS symptoms have been suggested yet [36,37]. Some nutritional interventions have been considered as appealing approaches, presenting a simple and quite decreased risk method to possibly recover adverse outcomes in individuals with mental disturbances to achieve the remission and improvement of clinical conditions, well-being, and life expectancy of individuals with MS [38,39,40].

Currently, only a few clinical studies have addressed the impact of nutritional interventions such as a low saturated fat diet in MS disease management, highlighting the strong demand to perform more research to understand the long-term effectiveness of dietary interventions in MS patients [41,42,43]. In a case–control study, a fish/seafood intake one time per week or one time per month with consistent fish oil treatment was related with a 44% lower disease risk or clinically isolated syndrome (CIS) compared to an intake of fish/seafood lower than one time per month and no fish oil supplement use [44]. Currently, only a few studies have evaluated the possible beneficial effect of healthy dietary patterns such as the Mediterranean diet (MD) on the disease disability and symptom severity of individuals with MS—studies which have suffered from several controversial and inconclusive results [45,46,47,48,49,50,51].

Notably, whether nutritional habits and lifestyle, such as the MD, may positively affect the disease progression are still unknown, while the currently available therapeutic approaches have not revealed any validated evidence on nutrition status and lifestyle [52,53]. Although the potential positive effects from any dietary pattern in MS have still not been proved, it is well-recognized that malnutrition may exacerbate MS symptoms’ severity [54,55]. Recently, we found that a higher MD compliance was significantly associated with a lower incidence of disease disability and symptom severity, depressive symptoms, anxiety behavior, perceived stress, inadequate sleep quality, cognitive impairment, and low physical activity levels in older individuals with MS [54,55]. Although there are currently several dietary patterns, including the MD, and specific food components with high bioactivity, which seem to slow down disease progression and improve MS symptoms, there are also some conflicting results, while most of the existing studies enrolled a small number of MS patients [56].

The pathophysiological pathways governing the MS course and the disability progression are very complex. Multisystem modifiable comorbidities are common in patients with MS, such as neurological disturbances, psychiatric and cardiovascular comorbidities (e.g., hypertension, dyslipidemia), long-term lung diseases, and metabolic disorders [57,58]. These modifiable comorbidities can enhance the complexity of MS patients’ support, presenting significant clinical and socioeconomic complications [57,58]. There are also diverse modifiable risk factors, such as obesity, lack of exercise, alcohol consumption, and smoking, which may increase the risk of the development and/or management of MS [57,58]. Both modifiable comorbidities and risk factors may differently affect the course and the progression of MS, highlighting the need for more personalized treatment approaches for each MS patient separately [59].

In view of the above considerations, the present cross-sectional study aims to investigate the potential effect of MD adherence in disease disability and symptoms’ severity, quality of life, physical activity levels, and depressive symptoms in an adequate population of 558 MS patients aged 18–64 years with no history of any severe chronic diseases except for MS. Several sociodemographic, anthropometric, and lifestyle factors were also evaluated by validated questionnaires, while a series of blood biochemical parameters were retrieved from MS patients’ medical records.

## 2. Materials and Methods

### 2.1. Study Population

Initially, 845 adults aged 18–64 years old were randomly enrolled from 8 different, geographically diverse Greek regions, urban, rural, and islands, Athens, Thessaloniki, Alexandroupolis, Larissa, Patra, Crete, and South and North Aegean. Recruitment to the study was performed between April 2016 and December 2020 in community-dwelling adults mostly by their visits in health care units and public or private hospitals.

**Figure 1:**
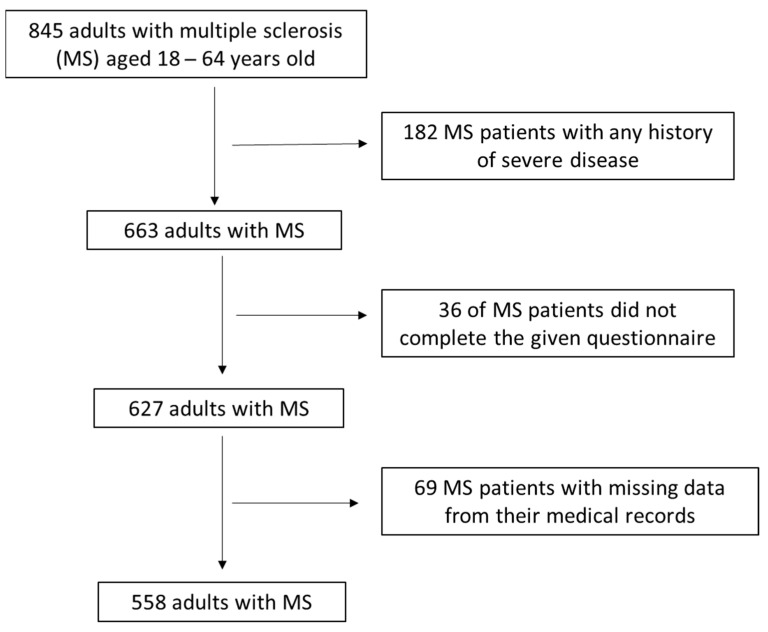
Flow chart diagram of study population enrolment.

During their thorough recruitment, 182 (21.5%) enrolled adults diagnosed with severe, untreated, chronic disease symptoms like cardiovascular diseases, cancer, metabolic disorders, autoimmune diseases, or neurodegenerative or mental health disorders were excluded from the survey. From the remaining 663 enrolled adults, 36 (5.4%) of them did not complete all the questions of the given questionnaire or interrupted the interview, while for another 69 (10.4%) MS patients there were some missing data from their medical records. Finally, 558 adults diagnosed with MS were included in the study with a final response equal to 66.0%. A flow chart diagram of study enrollment is depicted in Figure 1. Collectively, by applying the main inclusion criterion, the randomly enrollment of adults aged 18–64 years old were collected. As main exclusion criteria, the presence of any severe chronic disease, the non-completion of all questions of the given questionnaire, the interruption of the enrolled participants during interviews, and missing data from the participants’ medical records were applied.

All participants’ data were confidential, and all participants were informed of the aim of the study and signed a written consent form agreeing to publish their information anonymously. In our study, we followed all the guidelines of the Declaration of Helsinki and acted in agreement with the World Health Organization (52nd WMA General Assembly, Edinburgh, Scotland, 2000). The Ethics Committee of the University of the Aegean (ethics approval protocol: no 18/22.9.2016, agreement date: 22 September 2016) approved the design and the implementation of the present study, as well as the consent approval of the applicants.

### 2.2. Study Design

#### 2.2.1. Sociodemographic and Anthropometric Factors Assessment of the Study Population

Patients’ age, sex, educational level, economic level, nationality, and smoking habits were self-reported by face-to-face interviews of the participating patients with trained personnel. Economic status was classified according to annual income: Class 0 ≤ EUR 5000, Class 1 ≤ EUR 10,000, Class 2 ≤ EUR 15,000, Class 3 ≤ EUR 20,000, Class 4 ≤ EUR 25,000, and Class 5 > EUR 25,000, based on the per capita gross national product. Financial level was further categorized as low for an annual income ≤ EUR 10,000, intermediate for an annual income > EUR 10,000 and ≤EUR 20,000, and high for an annual income > EUR 20,000.

Trained personnel (e.g., doctors, physicians, and nurses, as well as nutritionists and dietitians) described in detail to the assigned individuals all the questions of the questionnaires to establish accurate responses. We implemented face-to-face interviews in which all interviewers directly communicated with the study participants in accordance with the prepared questionnaires to enhance the reliability of responses and to minimize recall bias. Trained personnel measured anthropometric indices, body mass index (BMI), and the waist and hip circumference ratio (WHR) as per protocol [52,53]. In fact, we measured the body weight of the participants using the identical electronic scale, and participants’ height and waist and hip circumference using a portable stadiometer (Chapter HM200P, Medi Shop, Athens Greece).

#### 2.2.2. Disease Disability, Physical Activity, Quality of Life, and Depression Assessment of the Survey Population

The Expanded Disability Status Scale (EDSS) was utilized to for the estimation of the disease disability of the enrolled individuals with MS. The Expanded Disability Status Scale (EDSS) is a validated and well-known method of quantifying disability in MS and monitoring changes in the level of disability over time [56]. EDSS Steps 1.0 to 4.5 refer to patients who are fully ambulatory, whereas EDSS Steps 5.0 to 9. are defined by the impairment to ambulation and standard equivalents and weakening to walking. The MS Quality of Life-54 (MSQOL-54), a well-recognized, validated questionnaire was applied for assessing the multidimensional health-related quality of life of individuals with MS [60,61]. Two summary scores—physical health and mental health—can also be derived from a weighted mixture of scale scores of MSQOL-54 [60,61]. Moreover, there are twelve subcategories: physical functioning, role limitations—physical, role limitations—emotional, pain, emotional well-being, energy, health perceptions, social function, cognitive function, health distress, total quality of life, and sexual functionality [60,61]. There are also two single element evaluates: happiness with sexual function and change in health [60,61].

Physical activity was assessed using the International Physical Activity Questionnaire (IPAQ) in which individuals report how much exercise they completed in a standard week. This self-administered questionnaire, utilized globally, assesses the total physical activity over the previous 7 days, to classify it as low, moderate, or high [62]. IPAQ methods have systematically been checked and showed adequate consistency and appropriate validity properties, at least as good as other self-responded PAQs [62]. The questionnaires were completed by qualified personnel (e.g., doctors, physicians, and nurses) as well as nutritionists and dietitians by face-to-face interviews with the enrolled individuals.

The Beck Depression Inventory (BDI-II) was used to assess the depression of the enrolled individuals. This questionnaire includes 21 groups of statements. It is a widely used psychometric test for evaluating the intensity of depression symptomatology [63]. BDI-II includes items associated with depression symptoms such as hopelessness and irritability, cognitions such as guilt or feelings of being punished, and symptoms such as fatigue, weight decrease, and low sexual interest [63]. The BDI-II is well-recognized as a greatly suitable psychometric tool, having sufficient reliability and capacity to distinguish between depressed and non-depressed people, and enhanced concurrent, content, and structural validity. Based on the available psychometric evidence, the BDI-II is a cost-effective questionnaire for evaluating the severity of depressive symptoms, with extensive applicability for research and clinical practice globally [63].

#### 2.2.3. Mediterranean Diet Adherence Assessment of the Study Population

MD compliance was evaluated by the Mediterranean Diet Score (MedDietScore) [64]. This is a food frequency questionnaire (FFQ) with 11 selected food groups according to the MedDietScore index [64]. Every question has six potential responses, marked from 0 to 5 points based on the degree of compliance for each food group. The sum of the 11 questions results in a score between 0 and 55; greater scoring represents greater MD compliance. For cereals, potatoes, fruits, vegetables, dairies, and olive oil, the consumption frequency is adjusted per day. For legumes, seafood, red meat, and poultry, the consumption frequency is adjusted per week [64]. The 11th question determines wine drinking daily with moderate drinking (≤1 and ≤2 drinks daily for women and men, respectively; one drink = 100 mL = 12 g ethanol) receiving the greatest score [64].

#### 2.2.4. Blood Circulating Biochemical Parameters of the Study Population

Blood circulating biochemical parameters’ levels including ferritin, albumin, creatinine, red blood count (RBC) hemoglobin (HMG), hematocrit, red cell distribution width (RDW), mean corpuscular volume (MCV), mean corpuscular hemoglobin (MCH), mean corpuscular hemoglobin concentration (MCHC), white blood count (WBC), neutrophils (NEUT), lymphocytes (LYMPH), monocytes (MONO), eosinophils (EO), basophils (BASO), and platelets (PLT) were recovered by the medical files of the assigned individuals with MS.

### 2.3. Statistical Analysis

Student’s *t*-test was applied for continuous variables following normal distribution, which was established by the Kolmogorov–Smirnov test. The chi-square test was used for categorical variables. The quantitative variables following normal distribution are expressed as the mean value ± standard deviation (SD) and the qualitative variables as absolute or relative frequencies. Multiple binary logistic regression was applied for assessing if MD compliance could independently be associated with disease disability, quality of life, physical activity, and depression after adjusting for possible confounders. As confounding factors, those covariates which were significantly associated with the MD in unadjusted analysis were included. Multiple binary logistic regression results are expressed as relative ratios (RR) and 95% confidence intervals (CI). Differences were recognized as significant at *p* < 0.05 and a 95% confidence interval. Statistica 10.0 software, Europe (Informer Technologies, Inc., Hamburg, Germany), was used to perform the statistical analysis of the study data.

## 3. Results

### 3.1. Descriptive Statistics of the Study Population

In Table 1, the descriptive statistics of the enrolled patients are depicted. The mean age of the assigned patients was 38.6 ± 12.2 years old (range: 18–64 years). Concerning patients’ sex, 75.3% were women and the remaining 24.7% were men. The mean number of years of education was 12.5 ± 4.9 years. Regarding economic status, 59.2% of the patients reported a low annual income, 29.0% of them had a medium annual income, and 11.8% of them documented a high annual income. As far as smoking habits are concerned, 27.8% of the assigned patients were regular smokers and 72.2% were never smokers at all. Most of the enrolled patients (81.7%) reported a Greek nationality and the remaining 18.3% reported other nationalities.

Amongst the enrolled patients with MS, 21.1% were classified as obese, 35.7% were categorized as overweight, and 43.2% exhibited a normal body weight status. Concerning the abdominal obesity expressed by WHR, 20.8% had a high WHR, 39.8% presented a moderate WHR, and 39.4% exhibited a normal WHR. Regarding the physical activity of the assigned adults with MS based on the IPAQ classification, 29.9% of the patients had low physical activity levels, 28.9% exhibited medium physical activity levels, and 41.2% had high physical activity levels.

As far as the Expanded Disability Status Scale (EDSS) classification is concerned, 45.2% of MS patients showed a score of 0–2.5, 29.6% had a score of 3.0–4.5, 4.5% showed a score of 5.0–6.5%, and 10.7% had a score ≥ 7.0. Based on the Multiple Sclerosis-Quality of Life Questionnaire (MSQOL-54) items’ classification, 49.1% of the assigned individuals with MS showed a score lower than the mean value and the remaining 50.9% of them had a score higher than the mean value. In total, 37.3% of the assigned MS patients showed depressive symptoms based on BDI-II. The mean values of the blood biochemical parameters are analytically depicted in Table 1.

### 3.2. Association of MD Compliance with Socio-Demographic and Anthropometry Parameters of the Study Population

In cross-tabulation, a higher MD adherence was not associated with any sociodemographic characteristic like patients’ age and sex, educational level, economic status, and nationality (Table 2, *p* > 0.05). Elevated MD compliance was significantly related with a decreased incidence of overweight and obesity amongst the patients suffering from MS (Table 2, *p* < 0.0001). Abdominal obesity was also considerably more frequently noted in patients presenting lower levels of MD compliance (Table 2, *p* = 0.0004).

### 3.3. Association of MD Compliance with Disease Disability, Quality of Life, Physical Activity Levels and Depressive Symptoms of the Enrolled MS Patients

The disease disability of the assigned MS patients indicated a strong association with their MD compliance (Table 2, *p* < 0.0001). In fact, MS patients presenting a higher MD compliance showed considerably decreased scores regarding EDSS classification (Table 2, *p* < 0.0001). On the contrary, patients with a very low or low MD compliance showed significantly higher scores based on EDSS classification (Table 2, *p* < 0.0001). Moreover, patients adhering to the MD at higher levels showed significantly greater levels of physical activity than patients with a lower MD adherence (Table 2, *p* < 0.0001). In addition, individuals with MS presenting a higher MD compliance had a significantly better quality of life related with MS symptomatology as assessed by MSQOL-54 (Table 2, *p* < 0.0001). On the contrary, patients with MS presenting very low or low MD compliance had a considerably worse quality of life related with MS symptomatology as assessed by MSQOL-54 (Table 2, *p* < 0.0001). In addition, a higher MD compliance was associated with a lower prevalence of depressive symptoms in patients with MS (Table 2, *p* = 0.0009).

**Figure 2:**
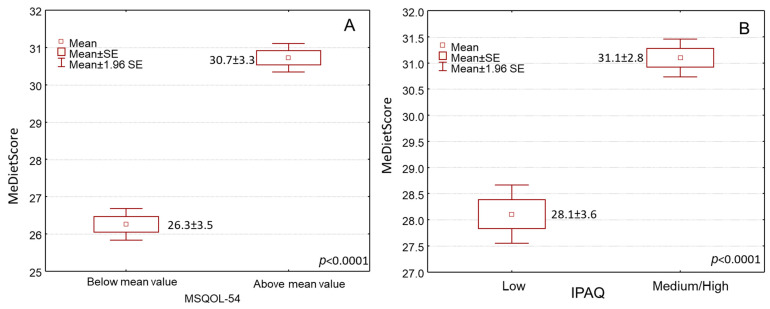
Box and whisker plots of MedDietScore with (**A**) depression and (**B**) the Expanded Disability Status Scale (EDSS).

**Figure 3:**
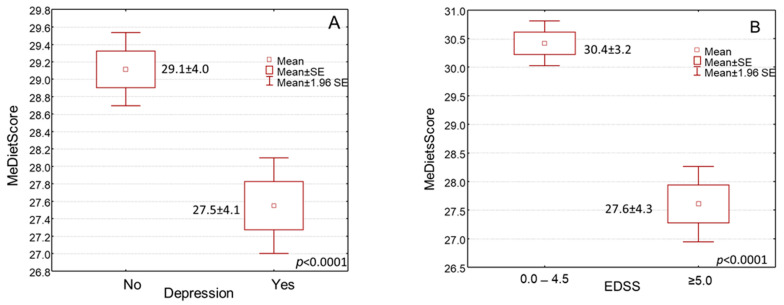
Box and whisker plots of MedDietScore with (**A**) MS Quality of Life-54 (MSQOL-54), and (**B**) International Physical Activity Questionnaire (IPAQ).

By treating MD compliance as a continuous variable (MedDietScore), we found that patients with higher levels of the MedDietScore were significantly associated with having no depressive symptoms compared to those with lower MedDietScore levels (Figure 2A, *p* < 0.0001). Accordingly, higher MedDietScore levels were significantly found in patients with reduced-disability MS disease (estimated by EDSS) than those with advanced-disability disease (Figure 2B, *p* < 0.0001). In addition, a greater MD compliance was considerably associated with a better quality of life estimated by the MSQOL-54 (Figure 3A, *p* < 0.0001). In addition, an elevated MedDietScore was considerably more frequently noted in MS patients with higher physical activity levels (Figure 3B, *p* < 0.0001).

### 3.4. Association of MD Compliance with Blood Biochemical Parameters of the Enrolled MS Patients

Lower levels of blood circulating ferritin, an indicator of iron deficiency, were significantly more frequently noted in patients with a decreased MD adherence (Table 2, *p* = 0.0084). Accordingly, lower levels of serum albumin, an indicator of malnutrition, were significantly more frequently noted in patients with a decreased MD compliance (Table 2, *p* = 0.0012). Reduced levels of blood circulating red blood cells (RBC), hemoglobin, and hematocrit, which are also indicators for iron deficiency, were considerably more frequently noted in patients presenting an elevated MD adherence (*p* = 0.0023, *p* = 0.0048 and *p* = 0.0059, respectively). Higher levels of blood circulating white blood cells (WBC), an indicator of inflammation, were considerably more frequently noted in patients presenting a decreased MD adherence (Table 2, *p* = 0.0343). All the other examined blood biochemical parameters did not exhibit any relation with MD compliance (Table 2, *p* > 0.5)

### 3.5. Multivariate Analysis of MD Compliance by Adjusting for Potential Confounders

In multivariate binary logistic regression analysis, we included only the parameters which exhibited a considerable relation with MD adherence in univariate statistical analysis. Specifically, MD compliance was independently associated with patients’ BMI and WHR, disease disability expressed by EDSS, physical activity classified by IPAQ, quality of life of MS patients categorized by MSQOL-54, depression assessed by BDI-II and blood circulating albumin, RBC, hemoglobin, and hematocrit levels (Table 3, *p* < 0.05).

In fact, patients with a lower MD adherence had a more than two-fold higher incidence of being affected by overweight or obesity (Table 3, *p* = 0.0018). Accordingly, patients adhering to the MD at lower levels showed a more than two-fold higher incidence of abdominal obesity (Table 3, *p* = 0.0102). Patients presenting a decreased MD adherence showed at a significantly independent level a two-fold higher prevalence of advanced disease disability (EDSS score ≥ 5.0) than those with lower disease disability (EDSS score ≤ 4.5) (Table 3, *p* = 0.0009). Accordingly, patients presenting a greater MD compliance had a 76% significantly higher incidence of elevated physical activity (Table 3, *p* = 0.0037). Moreover, patients with a greater MD adherence showed a 95% higher prevalence of a better quality of life related with MS symptomatology than those presenting a decreased MD adherence (Table 3, *p* = 0.0011). Furthermore, patients with a higher MD adherence had a two-fold lower incidence of depressive symptoms compared to MS patients with a decreased MD compliance (Table 3, *p* = 0.0107).

Patients with a higher MD adherence showed a 32% greater incidence of presenting blood circulating RBC over the mean value (Table 3, *p* = 0.0192). Accordingly, patients with a higher MD compliance exhibited a 47% greater frequency of presenting circulating hemoglobin levels over the mean value (Table 3, *p* = 0.0204). Patients with an elevated MD adherence had a 51% higher frequency of presenting a hematocrit over the mean value (Table 3, *p* = 0.0256). The other blood biochemical parameters (ferritin and WBC) did not remain significant in multivariate analysis (Table 3, *p* > 0.05).

## 4. Discussion

Malnutrition constitutes a severe issue, which negatively affects the prognosis of individuals with neurodegenerative diseases, including MS [28,29]. Malnutrition is negatively associated with disease severity, while the intake of nutrients has been shown to be decreased with disease progression in individuals with MS [65]. In a more recent study conducted on 147 MS patients, a deterioration in nutritional status was noted in 87.8% of the enrolled individuals with MS. Moreover, this study showed that 20% of participating patients were malnourished and 80% were at risk of malnutrition, while the proportion of patients with excess body mass was 46.8% [66]. A cross-sectional, multicenter survey performed on 353 MS patients also showed that nutritional habits and nutritional status may have a rather little impact on fatigue in MS patients [67]. On the other hand, a single-blinded, parallel-RCT conducted on 120 patients with MS clearly indicated that nutrition counseling and support may significantly improve anthropometric measurements, nutritional habits, nutrient intake, and adequacy [68]. Although the possible positive effects from any healthy nutritional pattern in MS have still not been completely confirmed, it has been well-recognized that malnutrition may potentially exacerbate MS symptoms’ severity [51,55].

In this aspect, we recorded that elevated MD compliance could exert beneficial effects against disease disability and symptoms’ severity in individuals with MS. More to the point, the present study has supported evidence that patients with higher MD compliance exhibited a more than 2-fold lower prevalence of being overweight or obese, also presenting a more than 2-fold lower frequency of abdominal obesity. Notably, lower MD adherence was associated with a 2-fold higher prevalence of advanced disease disability. Additionally, higher MD compliance was associated with elevated physical activity and a better quality of life in MS patients, while decreased MD compliance was associated with a higher prevalence of depressive symptoms and lower levels of certain blood biochemical parameters, which are indicators of iron deficiency, malnutrition, and inflammation.

In accordance with our study, there are currently a limited number of studies assessing the possible positive effect of healthy nutritional patterns like MD on the disease disability and symptom intensity of patients with MS; however, some of them were characterized by controversies and provided no conclusive results. In this aspect, many studies have established the protective effects of the MD against several chronic diseases, among which are diabetes, cardiovascular diseases, metabolic disorders, tumor malignancies, mental and psychiatric disorders, and aging disorders, and against overall mortality [69,70]. Also, the MD has been considered as a dietary pattern that may exert a protective effect against the neurodegenerative process, including MS, since this type of diet is rich in antioxidants, anti-inflammatory agents, fibers, and omega-3 polyunsaturated fatty acids [69,70]. Furthermore, substantial evidence has revealed that MD can promote a reduction in systemic inflammation in several human diseases, including MS [71]. Notably, several clinical studies in the last few years have supported the substantial evidence that most of the MD positive impacts may be highly associated with its anti-inflammatory and antioxidant effects, while its efficiency in preventing increases in waist circumference and obesity has further been established by several studies [72]. These beneficial impacts of the MD may mostly be attributed to its numerous ingredients, which can exert both anti-inflammatory and antioxidant properties [73]. Beyond the anti-inflammatory and antioxidant effects of the MD, this dietary pattern has also been associated with longer telomere length, slowing down human aging, and promoting a longer human lifespan [74].

In accordance with our results, a population-based case–control survey documented that the MD was related with a decreased probability of MS compared to a Western-type nutrition [42]. In the above study, the diet measures were enacted over the 5 years preceding disease onset, which would enable them to state causal relationships [42]. In line with our study, a retrospective survey conducted on 95 patients with MS also indicated that the MD was associated with the EDSS score and physical and well-being-related characteristics, supporting that the MD could be considered as an efficient dietary pattern that may be related with improved disability levels and a better quality of life of individuals with MS [43]. Also, a cohort survey performed on individuals with MS aged 18–65 indicated that MD alignment predicted lower objective and patient-reported disability [66]. Moreover, a multi-center, cross-sectional study performed on 478 patients presenting clinically definite MS demonstrated that a low adherence to the MD was higher in overweight patients compared to obese patients, even if MD adherence had no significant relationship with the levels of patients’ disability [44]. Another cross-sectional study conducted on 424 MS patients and 165 matched healthy individuals showed that MS patients exhibited a healthier dietary pattern overall compared to healthy individuals and that vegetables and seafood consumption were related with improved disability outcomes [45].

In agreement with our study, a more recent cross-sectional survey conducted on 102 MS patients documented that MD adherence was negatively associated with fatigue disease severity scores, even if it was not associated with MS-related symptoms [46]. It has also been indicated that a decreased intake of red meat, saturated fatty acids, and sweets, as well as an elevated seafood intake, was supported to attenuate MS symptomatology and fatigue intensity [46]. Moreover, a case–control study showed that the MD, including unprocessed red meat, was related with a lowered probability of an initial clinical diagnosis of CNS demyelination (FCD), a common precursor to MS, in Australian adults [67]. Notably, the addition of non-processed red meat to the MD was suggested to exert favorable effects in adults at a high risk of MS [75]. In contrast, an enhanced intake of ultra-processed foods was associated with an elevated probability of FCD in this Australian cohort [68]. Accordingly, a higher ultra-processed food intake was associated with moderate-to-high MS severity compared to a decreased intake in both unadjusted and adjusted analysis [76].

The present cross-sectional study has some strengths, as it was conducted on an adequate number of adults suffered from MS aged 18–64 years without any previous severe disease and who were assigned from eight geographically diverse regions of our country, containing both urban and rural regions. Thus, the study population is reasonably representative, as it was obtained from diverse regions of our country. This means that the generalizability of our findings could be applied to the MS patients of our country, but the results of our study cannot be generalized to other populations due to the presence of potential phylogenetic differences with the populations of other countries and continents. This study has also provided substantial evidence for the effect of MD on disease disability, quality of life, physical activity, and depressive symptoms, independently of several potential confounding factors that could influence the impact of the MD against MS. In fact, the use of several sociodemographic and anthropometric characteristics and blood biochemical parameters was not found to affect the association of the MD with disease disability, physical activity, quality of life, and depression in individuals diagnosed with MS. In addition, the present study utilized both BMI and WHR, which reflect both body weight status and the adiposity distribution. Moreover, both BMI and WHR were measured by experienced personnel and were not self-reported, avoiding recall biases. A last advantage of our study was the usage of qualified questionnaires like the EDSS, MSQOL-54, IPAQ, BDI-II, and MedDietScore, as well as the performance of face-to-face interviews with the MS patients to enhance the validity of their answers and to decrease recall bias.

However, it should be noted that the present study has reasonably been characterized by some limitations. The cross-sectional design of our study restricts the probability for etiological conclusions and has the potential for recall biases, specifically concerning self-reported questions even if we applied face-to-face interviews. More to the point, the cross-sectional design of our survey cannot support any causality effect of MD adherence with disease disability, symptoms’ intensity, quality of life, physical activity status, and depressive symptoms in adults presenting with MS. This could only be obtained by the performance of prospective studies in the future. Furthermore, although a thorough approach for confounding adjustment was applied, we recognize the possibility of additional undetermined confounding factors. Even if we performed adjustment for several confounding factors, it remains a probability that residual confounding could also influence our results, such as several other mental health disturbances of participants beyond depression (e.g., anxiety, stress, bipolar disorder, schizophrenia, personality disorders, etc.). Eating disorders like anorexia nervosa, bulimia nervosa, binge eating disorder, and emotional eating may also exert a confounding effect. In this context, mental health disturbances are common comorbidities of MS, with depression being the main one, while anxiety, stress, and eating disorders with a psychiatric background may also exhibit a confounding effect [77,78]. Although these symptoms are a crucial factor affecting the quality of life in MS, they are often overlooked and undertreated [79,80,81]. However, future clinical studies should be performed by including all of the above potential confounding factors for more precise conclusions to be drawn. An additional limitation of our study concerns the fact that we did not record whether the enrolled participants are receiving at the time of study any medication or treatment for a non-chronic disease such as cold, fever, catatonia, nausea, or some other short-term disease. Thus, drug prescriptions could be a considerable confounder, which must be considered in the forthcoming studies. Moreover, as mentioned and justified above, the generalization of our study could be applied only to the MS patients of our country. In addition, the high waves of immigration from the Asian and African countries to Europe could be considered as an additional confounder, which will be a challenge for future research studies. Lastly, as the prevalence of MS disease concerns mainly women, future studies should also be performed by examining only women to avoid the potential confounding impact of sex and the related hormones of the enrolled patients.

It should be noted that due to the complicated nature of MS disease, novel personalized diagnosis and management approaches can more effectively help patients and clinicians plan future treatments. In this aspect, novel computational models are at the forefront of the pursuit of personalized medicine due to their promising descriptive and predictive abilities [82,83]. Notably, in the presence of complex and heterogeneous data, as happened in MS disease, patient stratification is a strong prerequisite for effective personalized medicine, as disease development is frequently driven by individual variability and unpredictable environmental events [82,83]. Thus, the use of personalized medicine to develop novel therapeutic strategies tailored to the individual patients’ characteristics and the specific disease activity features, as well as to the patients’ needs and preferences, may bring about novel challenges and perspectives [59,82,83].

## 5. Conclusions

This cross-sectional study is one of the few available studies which has provided clinical evidence that elevated MD compliance could be related with a decreased disease disability symptoms’ intensity, as well as an improved quality of life, a higher physical activity status, and fewer depressive symptoms in individuals with MS. Future well-organized MD interventional studies are strongly recommended to assess more efficiently the probable favorable impact of the MD in adults with MS. Overall, an appropriate and balanced diet such as the MD could be exceptionally helpful in promoting the well-being of individuals with MS, and effectively supporting therapeutic approaches. Prospective studies are essential to establish whether there is a causal effect between the MD and an MS improvement in disorder disability and symptom intensity. Future strategies and policies could advise and support the general population and especially the individuals with MS concerning the beneficial effects of the MD to prevent human disorders and to improve or even slow down the disease disability and symptom severity of MS, as well as MS-related comorbidities. Moreover, future studies should take into account as confounding factors the potential mental health disturbances of MS patients, which are common comorbidities in MS beyond depression.

## Figures and Tables

**Table 1:** Descriptive statistics of the study population.

Characteristics (*n* = 558)	Descriptive Statistics
Age (mean ± SD; years)	38.6 ± 12.2
Gender (*n*, %)	
Female	420 (75.3%)
Male	138 (24.7%)
Education level (mean ± SD; years)	12.5 ± 4.9
Family economic status (*n*, %)	
Low	330 (59.2%)
Medium	162 (29.0%)
High	66 (11.8%)
Smoking habits (*n*, %)	
No smokers	403 (72.2%)
Regular smokers	155 (27.8%)
Nationality (*n*, %)	
Greek	456 (81.7%)
Other	102 (18.3%)
BMI status (*n*, %)	
Normal weight	241 (43.2%)
Overweight	199 (35.7%)
Obese	118 (21.1%)
WHR (*n*, %)	
Low	220 (39.4%)
Moderate	222 (39.8%)
High	116 (20.8%)
IPAQ (*n*, %)	
Low	167 (29.9%)
Medium	161 (28.9%)
High	230 (41.2%)
EDSS (*n*, %)	
0–2.5	252 (45.2%)
3.0–4.5	165 (29.6%)
5.0–6.5	81 (14.5%)
≥7.0	60 (10.7%)
MSQOL-54 (*n*, %)	
Below mean value	274 (49.1%)
Over mean value	284 (50.9%)
Depression (*n*, %)	
Undepressed	350 (62.7%)
Depressed	208 (37.3%)
Ferritin (mean ± SD; ng/mL)	118 ± 54.3
Albumin (mean ± SD; g/dL)	3.8 ± 0.7
Creatinine (mean ± SD; mg/dL)	0.8 ± 0.5
RBC (mean ± SD; 10^6^/mL)	4.4 ± 0.5
Hemoglobin (mean ± SD; g/dL)	14.0 ± 4.6
Hematocrit (mean ± SD; %)	37.8 ± 5.5
RDW (mean ± SD; fL)	58.3 ± 7.6
MCV (mean ± SD; fL)	87.8 ± 8.5
MCH (mean ± SD; pg)	29.5 ± 4.4
MCHC (mean ± SD; g/dL)	34.1 ± 4.3
WBC (mean ± SD; k/μL)	7.5 ± 2.4
NEUT (mean ± SD; %)	67.5% ± 9.6
LYMPH (mean ± SD; k/μL)	34.1% ± 4.7
MONO (mean ± SD; %)	6.5% ± 1.8
EO (mean ± SD; %)	4.0% ± 1.7
BASO (mean ± SD; %)	0.5% ± 0.5
PLT (mean ± SD; k/μL)	278.0 ± 12.9
Vitamin B12 (pg/mL)	585 ± 44.7

IPAQ: International Physical Activity Questionnaire, EDSS: Expanded Disability Status Scale, MSQOL-54: Multiple Sclerosis—Quality of Life Questionnaire 54 items, RBC: red blood count, RDW: red cell distribution width, MCV: mean corpuscular volume, MCH: mean corpuscular hemoglobin, MCHC: mean corpuscular hemoglobin concentration, WBC: white blood count, NEUT: neutrophils, LYMPH: lymphocytes, MONO: monocytes, EO: eosinophils, BASO: basophils, PLT: platelets.

**Table 2:** Association of MD compliance with socio-demographic and anthropometry parameters, disease disability, quality of life, physical activity, and blood biomarkers.

Characteristics (*n* = 558)	Mediterranean Diet Adherence				
	Very Low 138 (24.7%)	Low 138 (24.7%)	Moderate 141 (25.3%)	High 141 (25.3%)	*p*-Value
Age (mean ± SD; years)	38.9 ± 11.9	38.3 ± 11.3	38.5 ± 12.1	38.8 ± 12.2	*p* = 0.5678
Sex (*n*, %)					*p* = 0.6636
Female	102 (73.9%)	108 (78.3%)	102 (72.3%)	108 (76.6%)	
Male	36 (26.1%)	30 (21.7%)	39 (27.7%)	33 (23.4%)	
Educational status (mean ± SD; years)	12.2 ± 4.2	12.6 ± 4.8	12.4 ± 4.5	12.7 ± 4.7	*p* = 0.3121
Family economic status (*n*, %)					*p* = 0.6873
Low	81 (58.7%)	81 (58.7%)	87 (61.7%)	81 (57.4%)	
Medium	36 (26.1%)	45 (32.6%)	39 (27.7%)	43 (29.8%)	
High	21 (15.2%)	12 (8.7%)	15 (10.6%)	18 (12.8%)	
Smoking habits (*n*, %)					*p* = 0.8653
No smokers	96 (69.6%)	102 (73.9%)	102 (72.3%)	103 (73.0%)	
Smokers	42 (30.4%)	36 (26.1%)	39 (27.7%)	38 (27.03%)	
Nationality (*n*, %)					*p* = 0.1149
Greek	212 (84.1%)	136 (82.42%)	63 (77.8%)	45 (75.0%)	
Other	40 (15.9%)	29 (17.6%)	18 (22.2%)	15 (25.0%)	
BMI status (*n*, %)					*p* < 0.0001
Normal weight	48 (34.8%)	27 (19.6%)	89 (63.2%)	77 (54.6%)	
Overweight	45 (32.6%)	73 (52.9%)	25 (17.7%)	56 (39.7%)	
Obese	45 (32.6%)	38 (24.5%)	27 (19.1%)	8 (5.7%)	
WHR (*n*, %)					*p* = 0.0004
Low	54 (39.1%)	36 (26.1%)	61 (43.3%)	69 (48.9%)	
Moderate	46 (33.3%)	63 (45.6%)	53 (37.6%)	60 (42.6%)	
High	38 (27.5%)	39 (28.3%)	27 (19.1%)	12 (8.5%)	
IPAQ (*n*, %)					*p* < 0.0001
Low	99 (71.7%)	43 (31.2%)	10 (7.1%)	15 (10.6%)	
Medium	30 (21.8%)	75 (54.3%)	25 (17.7%)	31 (22.0%)	
High	9 (6.5%)	20 (14.5%)	106 (75.2%)	95 (67.4%)	
EDSS (*n*, %)					*p* < 0.0001
0–2.5	15 (10.9%)	39 (28.3%)	105 (74.5%)	93 (66.0%)	
3.0–4.5	57 (41.3%)	51 (37.0%)	24 (17.0%)	33 (23.4%)	
5.0–6.5	33 (23.9%)	33 (23.9%)	6 (4.3%)	9 (6.4%)	
≥7.0	33 (23.9%)	15 (10.9%)	6 (4.3%)	6 (4.3%)	
MSQOL-54 (*n*, %)					*p* < 0.0001
Below mean value	121 (87.7%)	103 (74.6%)	27 (19.1%)	23 (16.3%)	
Over mean value	17 (12.3%)	35 (25.4%)	114 (8.9%)	118 (83.7%)	
Depression (*n*, %)					*p* = 0.0009
Undepressed	66 (47.8%)	85 (61.6%)	96 (68.1%)	103 (73.0%)	
Depressed	72 (52.2%)	53 (38.4%)	45 (31.9%)	38 (27.0%)	
Ferritin (mean ± SD; ng/mL)	101.1 ± 52.1	112 ± 53.7	134 ± 53.5	136 ± 53.9	*p* = 0.0084
Albumin (mean ± SD; g/dL)	3.5 ± 0.7	3.6 ± 0.5	3.7 ± 0.6	4.1 ± 0.7	*p* = 0.0012
Creatinine (mean ± SD; mg/dL)	0.9 ± 0.6	0.7 ± 0.5	0.8 ± 0.4	0.9 ± 0.2	*p* = 0.3295
RBC (mean ± SD; 10^6^/mL)	4.1 ± 0.5	4.3 ± 0.4	4.6 ± 0.4	4.8 ± 0.3	*p* = 0.0023
Hemoglobin (mean ± SD; g/dL)	13.1 ± 3.2	13.8 ±4.4	14.2 ±3.1	14.9 ±2.9	*p* = 0.0048
Hematocrit (mean ± SD; %)	36.2 ± 5.6	37.3 ± 5.6	38.5 ± 5.4	39.1 ± 5.2	*p* = 0.0059
RDW (mean ± SD; fL)	57.9 ± 7.8	58.5 ± 7.3	58.1 ± 7.5	59.1 ± 7.2	*p* = 0.2895
MCV (mean ± SD; fL)	87.1 ± 8.3	88.0 ± 8.2	87.9 ± 8.3	87.9 ± 8.1	*p* = 0.2736
MCH (mean ± SD; pg)	28.8 ± 4.1	29.4 ± 4.2	29.7 ± 4.5	29.9 ± 4.6	*p* = 0.1943
MCHC (mean ± SD; g/dL)	33.6 ± 4.8	34.5 ± 4.6	34.3 ± 4.1	34.4 ± 4.2	*p* = 0.1291
WBC (mean ± SD; k/μL)	7.8 ± 2.1	7.6 ± 1.8	7.5 ± 2.3	7.3 ± 1.9	*p* = 0.0343
NEUT (mean ± SD; %)	67.8% ± 8.9	68.1% ± 9.7	67.4% ± 9.5	67.2% ± 9.2	*p* = 0.2897
LYMPH (mean ± SD; k/μL)	34.9 ± 4.8	34.5 ± 4.9	33.9 ± 4.5	34.2% ± 4.8	*p* = 0.2492
MONO (mean ± SD; %)	6.6% ± 1.7	6.3% ± 1.5	6.8% ± 1.9	6.5% ± 1.4	*p* = 0.4492
EO (mean ± SD; %)	3.9% ± 1.4	4.0% ± 1.6	3.9% ± 1.4	4.1% ± 1.1	*p* = 0.3736
BASO (mean ± SD; %)	0.5% ± 0.4	0.6% ± 0.5	0.6% ± 0.3	0.5% ± 0.4	*p* = 0.5738
PLT (mean ± SD; k/μL)	277.8 ± 12.6	278.4 ± 12.7	277.9 ± 12.8	278.7 ± 12.5	*p* = 0.3955
Vitamin B12 (pg/mL)	584 ± 42.3	585 ± 44.9	583 ± 45.3	586 ± 42.7	*p* = 0.4583

IPAQ: International Physical Activity Questionnaire, EDSS: Expanded Disability Status Scale, MSQOL-54: Multiple Sclerosis—Quality of Life Questionnaire 54 items, RBC: red blood count, RDW: red cell distribution width, MCV: mean corpuscular volume, MCH: mean corpuscular hemoglobin, MCHC: mean corpuscular hemoglobin concentration, WBC: white blood count, NEUT: neutrophils, LYMPH: lymphocytes, MONO: monocytes, EO: eosinophils, BASO: basophils, PLT: platelets.

**Table 3:** Multivariate analysis of MD compliance by adjusting for possible confounders.

Characteristics	Mediterranean Diet Adherence (Very Low + Low vs. Moderate + High)	
	RR * (95% CI **)	*p*-Value
BMI (overweight + obesity/normal weight)	2.13 (1.91–2.34)	*p* = 0.0018
WHR (moderate + high/low)	2.04 (1.71–2.38)	*p* = 0.0102
IPAQ (low/medium + high)	1.76 (1.48–2.02)	*p* = 0.0037
EDSS (≥5.0/≤4.5)	2.26 (2.06–2.37)	*p* = 0.0009
MSQOL-54 (below/over mean value)	1.95 (1.71–2.19)	*p* = 0.0011
Depression (undepressed/depressed)	2.08 (1.79–2.31)	*p* = 0.0107
Ferritin (below/over mean value)	1.87 (1.42–2.37)	*p* = 0.0746
Albumin (below/over mean value)	1.61 (1.29–1.91)	*p* = 0.0293
RBC (below/over mean value)	1.32 (0.97–1.79)	*p* = 0.0192
Hemoglobin (below/over mean value)	1.47 (1.05–1.89)	*p* = 0.0204
Hematocrit (below/over mean value)	1.51 (1.08–1.93)	*p* = 0.0256
WBC (over/below mean value)	1.19 (0.68–1.87)	*p* = 0.2983

* RR: relative ratio; ** CI: confidence interval.

## Data Availability

Research data are available upon request to the corresponding author.
